# Effect of Differentiated Relative Humidity of Air on the Quality of Traditional Speciality Guaranteed “Krakowska Sucha Staropolska” Sausage

**DOI:** 10.3390/foods11060811

**Published:** 2022-03-11

**Authors:** Marta Chmiel, Lech Adamczak, Dorota Pietrzak, Tomasz Florowski, Anna Florowska

**Affiliations:** Department of Food Technology and Assessment, Institute of Food Sciences, Warsaw University of Life Sciences—SGGW, 166 Nowoursynowska Street, 02-787 Warsaw, Poland; marta_chmiel@sggw.edu.pl (M.C.); dorota_pietrzak@sggw.edu.pl (D.P.); tomasz_florowski@sggw.edu.pl (T.F.); anna_florowska@sggw.edu.pl (A.F.)

**Keywords:** relative air humidity, “krakowska sucha staropolska” sausage, quality, traditional speciality guaranteed, drying

## Abstract

The aim of the study was to determine the effects of air relative humidity (RH: 60 and 80%) during the drying process of “krakowska sucha staropolska” (KSS) sausages on selected quality characteristics. The composition and production process of KSS sausages complied the requirements of traditional specialities guaranteed. It was found that the use of lower RH of drying air allowed a time reduction of 20%. Lowering the RH of air during the drying process did not affect the cross-sectional colour and odour of the sausages, colour components, TBARS values, protein, fat and salt content. However, the acceleration of the drying process resulted in very dried outer layers and less dry interior of KSS sausages. As a result, the sausages had lower scores in the sensory evaluation of hardness and overall acceptability. For this reason, shortening the drying process of sausages by using air with lower relative humidity is not recommended.

## 1. Introduction

All European countries, including Poland, have cultural traditions related to specific food products. Consumers, especially in European countries, have shown a new interest in traditional foods [1,2,3,4]. The high desirability of such products is influenced by their traditional character and reputation that has been built over many years. Desirability is related, among other things, to the unique taste and flavour of the products and their “naturalness” resulting from the limited use of food additives [5,6,7,8].

One of traditional meat product is “krakowska sucha staropolska” sausage (KSS sausage). The process of its production was described in 1926 [9]. Since 2018, this sausage has been registered in the European Union as a product of the Traditional Specialty Guaranteed (TSG) under the number TSG-PL-02145-14.6.2016. KSS sausage is a sausage made of pork and/or beef, coarsely ground, with a bar length of about 300 mm and a diameter of 50–70 mm, which can be produced in the natural intestines or in a protein casing. The surface of the sausage is dark brown, shiny and slightly wrinkled. On the cross-sectional area of the sausage, there are visible larger pieces of lean meat that are pink to dark pink in colour and smaller pieces of fat surrounded by the meat mass. A characteristic feature of KSS sausage is the taste of cured, smoked and roasted meat, with a distinct hint of pepper and an aftertaste of garlic and nutmeg [9].

One of the most important technological processes responsible for the quality of KSS sausages is the drying process. According to the specification TSG, KSS sausages should be dried to the desired yield, i.e., not more than 70% [9]. Drying is one of the oldest methods of food preservation. Currently, the drying process is used in the production technology of traditional meat products such as beef jerky in the USA [10,11] and similar jerky products such as pemmican in North American Artic, charque in Brazil [12], pastirma in Turkey [13], biltong in South Africa [14,15] and cecina in Spain [16]. In the meat industry in Poland, some meat products are also dried, such as “krakowska” sausage, “jałowcowa”/juniper sausage or kabanos sausages. During drying, complex physicochemical processes occur, which are related not only to the reduction of water content (while increasing in the content of other ingredients) in the product, but also to the formation of its characteristic sensory properties [17].

The drying process not only affects the quality of sausages but is also an important step in determining the economic results of production, as it is one of the most time- and energy-consuming stages in the production of dry sausage. According to Abdallah et al. [18] and Ojha et al. [19], the production of both modern and traditional products should now be focused on improving quality and stability and shortening the processing cycle. The duration of the drying process depends, among other things, on the parameters of the drying air, especially the relative humidity (RH) and temperature of air [20]. During the drying process of meat products, the RH of the air is often between 60 and 80% [21]. However, increasing the drying rate can lead to uneven drying of the product and the formation of an undesirable and overdried top layer (“crust”) on the surface of the sausages, which reduces their quality [22]. This problem is particularly observed in the rapid drying of products that are large in diameter.

The aim of the study was to determine the effects of differentiated relative air humidity (60 and 80%) on the drying process and the quality of traditional “krakowska sucha staropolska” sausage produced according to the production process described in the specification number TSG-PL-02145-14.6.2016 [9].

## 2. Materials and Methods

### 2.1. “Kakowska Sucha Staropolska” Sausage Production and the Layout of the Experiment

“Krakowska sucha staropolska” sausages (KSS sausages) are produced from pre-cut (pieces with a diameter of about 50 mm) and cured (72 h under refrigeration conditions, 4–6 °C) meat and fat raw materials, according to the recipe presented in Table 1.

The cured meat and fat were ground in a Mesko WN60 laboratory mincer (Mesko-AGD, Skarżysko-Kamienna, Poland). Meshes with a hole diameter of 8 mm for class IIA meat and fat (frozen to −4 °C) and 3 mm for class III meat were used. Class I meat was used directly after pre-cutting, i.e., in pieces with a diameter of 50 mm. The meat was mixed with the addition of water in Kenwood Major mixers (Kenwood, Havant, UK), under cooling conditions, for 35 min. Then, the spices and the frozen ground fat were added and the whole batter was mixed until the meat batter ingredients were evenly distributed (about 5 min). The prepared meat batter was filled into protein casings ø 55 mm with a manual Dick stuffer (Friedr, Dick GmbH&Co. KG, Deizisau, Germany), then bars were formed into 30 cm and clipped. The prepared bars were weighed to determine the yield of thermal treatment and cooling, and then hung on smoking sticks and settled at room temperature for about 2 h. After the settling phase, the sausages were thermally treated in a Jugema (Jugema, Środa Wielkopolska, Poland) smoking–steaming chamber. This process included surface drying, smoking with warm smoke at 55 °C for 30 min, steaming at 75 °C to a temperature of 60 °C at the geometric centre, and baking at 85 °C to a temperature of 70 °C at the geometric centre of the product. After thermal treatment, the sausages were moved to a cooling room (4–6 °C) and cooled for 24 h. After this time, the sausages were weighed again. KSS sausages were produced in three independent experimental repetitions from meat obtained from three different production batches, and 15 bars were produced in each repetition.

The sausages divided into two groups were dried in a drying chamber (PHU Chłodnictwo, Warsaw, Poland) at two different relative humidities (RH) of 60 ± 2% and 80 ± 2% and the same temperature 15 ± 2 °C. The relative air humidity and the temperature in the chambers were controlled by loggers (EL-USB-2 models, Lascar Electronics Ltd., Erie, PA, USA). The drying process of KSS sausages was carried out until they obtained the final yield required by the TSG specification, i.e., below 70% [9]. The yield of the sausages was monitored at 24 h intervals.

Due to the large diameter of the sausage bars (55 mm), it was assumed that the drying process (determined by the change in water content of the sausages) would run unevenly along their cross-sectional area. For this reason, the water activity was measured every 24 h and the water content was determined at different points of the slice (20 mm thick) cut from the sausages, dried at both relative humidities. For this purpose, three samples were cut from the slices using grip corks: an inner cylinder with a diameter of about 10 mm, a middle toroid with a diameter of about 20 mm and an outer toroid forming the remaining part of the slice. In the samples obtained this way, the water activity (aw) was measured, and the water content was determined (after grinding each part).

In addition, before and after the drying process, from each production batch, from the sausages dried at both relative air humidities, a sausage bar was collected for other determinations. In the tested sausages, the L*, a*, b* colour components were measured on the cross-sectional area of the bar, and then, after grinding the sausages (laboratory grinder Zelmer Diana 886.8, Zelmer, Rzeszów, Poland, mesh hole diameter 3 mm), the content of basic chemical components (water, protein, fat and salt) and TBARS index were determined. In addition, the sensory quality of the KSS sausages was evaluated.

### 2.2. Methods

#### 2.2.1. KSS Sausages Yield at Different Stages of the Production Process

The yield of thermal treatment and cooling, as well as the yield every 24 h of the KSS sausages drying process at RH60% or RH80%, were determined. These yields were determined in relation to the weight of the sausages before thermal treatment.

#### 2.2.2. Measurement of Water Activity (aw)

Water activity measurements were performed using an Aqua Lab CX-2 (Decagon Devices INC., Pullman, WA, USA). Measurements were carried out at a temperature of 25 ± 1.5 °C.

#### 2.2.3. Content of the Basic Chemical Components

Water, protein and fat content in KSS sausages were determined according to AOAC [23]. The salt content was determined using the potentiometric method according to PN-ISO 1841-2:2002 [24], using a 702 SM Titrino (Metrohm AG, Herisau, Switzerland).

#### 2.2.4. Measurement of Colour Components in the L*, a*, b* Scale

Colour components L*, a* and b* were measured with Minolta CR-200 (Konica Minolta, Wrocław, Poland, light source D65, observer 2°, measuring head hole 8 mm) calibrated according to the white standard (L* 97.81, a* −0.45, b* +1.88). The measurements were made each time in 5 repetitions on the cross-section area of the sausage bars, taking the average as the result of the determination.

#### 2.2.5. TBARS Index Determination

The values of reactive substances of thiobarbituric acid (TBARS) were determined according to the method of Shahidi [25]. The absorbance was measured with a spectrophotometer (Hitachi U-1100; Gemini bv., Apeldoorn, The Netherlands) at 532 nm against a blank containing 5 mL of 2-thiobarbituric acid (TBA) and 5 mL of 10% trichloroacetic acid (TCA). A constant coefficient of 2.34 was used for converting the absorbance units into TBARS values. These values were expressed as mg malondialdehyde per kg of sample (mg MAD/kg).

#### 2.2.6. Sensory Quality Evaluation

The sensory quality of KSS sausages after the drying process was evaluated using a hedonic scale (where 0 points corresponded to unacceptable and a 10-point evaluation as very desirable) for sensory characteristics such as cross-sectional area colour, aroma, taste, hardness and overall acceptability. The influence of RH of air on the sensory quality of KSS sausages was assessed after 96 and 120 h of drying. The sensory evaluation was carried out in six independent sessions, corresponding to three experimental repetitions and drying times (96 and 120 h). Each time, the evaluation was conducted by a 10-person trained panel. The panellists were assessed in terms of sensory sensitivity thresholds, and before the first session were trained to evaluate KSS sausages according to Polish Standards (PN-ISO 4121:1998) [26]. The KSS sausages were conditioned at room temperature for one hour before evaluation. As samples for sensory evaluation, slices of about 3 mm thick cut from bars were used. Sausage samples were placed on white plates, and a single sample consisted of slices of sausage dried at a specified RH of air and after the specified drying time. Each product was analysed in two independent replications, so each time the mean values were based on 20 individual results. Water was provided for the panellists to rinse their mouths between evaluations.

#### 2.2.7. Statistical Analysis

The results were statistically analysed using the Statistica program ver. 13PL (StatSoft, Inc., Tulsa, OK, USA). The influence of the relative air humidity during the drying process on the quality features of KSS sausages as yield, water activity, water content, the content of basic chemical components, L*, a* and b* colour components, as well as sensory characteristics, was determined. For this purpose, the One-way ANOVA analysis of variance and Tukey’s detailed HSD test at the significance level α = 0.05, or when comparing two averages, the Student’s *t*-test were used. The same statistical methods were used to determine the influence of drying time (at a certain relative humidity) on the above quality characteristics of the KSS sausages. In addition, for the water activity and water content of KSS sausages, the influence of the place where the sample was cut out from the sausage slice was assessed for given relative humidity of the drying air. The influence of relative air humidity on water activity and water content at a given drying time was also analysed. For this purpose, the Student’s *t*-test was used.

## 3. Results and Discussion

### 3.1. Yield of “Krakowska Sucha Staropolska” Sausages

Yield of KSS sausages on various stages of production are presented in Table 2. The yield of the sausages after heat treatment and 24 h of cooling was 98.8%. It was found that both the relative air humidity and the drying time had a significant (*p* ≤ 0.05) impact on the yield of sausages.

As assumed, sausages dried with air of a lower RH, i.e., 60%, were characterized by significantly lower (*p* ≤ 0.05) yield at each stage of the drying process. Moreover, it was found that after each 24 h of the drying process, both at RH60% and RH80%, there was a significant (*p* ≤ 0.05) decrease in product yield. In the case of sausages dried in air with RH80%, assumed by the TSG specification final product yield (below 70%), after 120 h of drying it was 68.0%. In contrast, sausages dried in air with RH60% achieved a yield below that assumed by the TSG specification, i.e., after 96 h of the process. For the sausages dried in this way, the process was continued for the next 24 h in order to compare their quality with sausages dried with in air with RH80%. The dynamics of changes in the yield of KSS sausages dried at 60% RH decreased significantly along with the extension of the drying time. Initially, i.e., after 24 h of the process, the yield decreased by 11.2 of % units, and in the final stage, i.e., between 96 and 120 h of drying, by only 1.6 of % units. When drying sausages at relative humidity of 80%, the dynamics the changes in the yield was more uniform, ranging from 5.0 to 7.2% units for each of the following 24 h of the drying process (Table 3). These differences could be caused by the initial significant dehydration of the surface of the sausages dried at 60% RH. According to Collell et al. [22], the product should be dried gradually during the drying process, as too rapid drying may result in an undesirable over-dried layer on the surface of the bars. Additionally, Arnau et al. [27] report that the use of low RH of air during drying promotes the formation of a crust on the surface, especially in the case of large-sized products such as dry-cured hams. It should be emphasized, however, that the use of lower RH of air in the KSS sausages drying process allowed for its reduction by 24 h, i.e., by 20%, which may be important for the economics of production. Chmiel et al. [28] indicate similar trends in the impact of relative air humidity on the yield in the drying process. The authors who studied the dynamics of the drying process of kabanos sausages found that lowering the humidity from 80 to 60% can reduce the drying time by up to 50%. This indicates that the possibility of reducing the drying time depends on the type of product, mainly its diameter.

### 3.2. Changes in the Water Activity of KSS Sausages

The drying process decreases the water activity, which determines the shelf life of the dried product [27,28,29,30,31]. The changes in water activity of “krakowska sucha staropolska” sausages during the drying process are presented in Table 3. The water activity of the KSS sausages before the drying process was from 0.970 to 0.976. With the increase in the drying time, both at 60% and 80% air RH, the water activity decreased, regardless of the location where the sample was cut out. A significant (*p* ≤ 0.05) decrease in water activity of KSS sausages dried with air at RH60% and RH80%, compared to the initial water activity (time 0 h) was observed after 120 h of drying for inner cylinders (0.950 and 0.953, respectively) and middle toroids (0.945 and 0.953, respectively) and after 96 h of drying for outer toroids (0.936 and 0.942, respectively). In the case of outer toroids, the water activity did not change significantly (*p* > 0.05) after a further 24 h of drying. Therefore, drying KSS sausages with air with different RH does not differentiate the rate of changes in water activity.

Significant differences in water activity between the places where the samples were cut out were found only after 120 h of the drying process, both with air with RH60% and RH80% (Table 3). The outer toroid samples had significantly lower (*p* ≤ 0.05) water activity than the middle toroid and the inner cylinder samples. This was due to faster removal of water from the outer layers of the product and limited diffusion of water from the inner layers [32]. However, no significant (*p* > 0.05) influence of air RH on water activity was found for the same sample cutting place, regardless of the drying time (Table 3). Arnau et al. [27] observed a similar dependence of the influence of RH on water activity in dry cured hams.

### 3.3. Changes in the Water Content in KSS Sausages

The water content in KSS sausages before the drying process was significantly different (*p* ≤ 0.05) depending on the place where the sample was cut out. Already at the beginning of the drying process, the samples of the outer toroid had significantly (*p* ≤ 0.05) lower water content then samples of the inner cylinder (Table 3). With the extension of the drying time, both at 60% and 80% RH of air, the water content decreased, regardless of the place where the sample was cut out. Significant (*p* ≤ 0.05) differences were observed in the KSS sausages dried in air at relative humidity of 80% compared to the initial water content from 48 h of drying, regardless of where the sample was cut out. For KSS sausages dried with air with RH80%, irrespective of the place where the sample was cut out, significant (*p* ≤ 0.05) differences in comparison with the initial water content were observed starting from 48 h of drying. The above observations show that the water loss from the outer layers of the sausages was faster (by 24 h) when dried with air at lower relative humidity. It was also found that, regardless of RH and the drying air and the locations where the samples were cut from the sausages, the water content decreased significantly within 96 h of the drying process. Extending the drying process for an additional 24 h did not result in significant changes in water content (Table 3).

As mentioned above, significant (*p* ≤ 0.05) differences in water content were found between the locations where the samples were excised, even before the drying process (Table 4). The samples of the outer toroid were characterized by a significantly lower (*p* ≤ 0.05) water content compared to the samples of the middle toroid and the inner cylinder. This means that the 24 h cooling of the sausages before the drying process did not equalize the water content in the entire volume of the bars, which weredifferentiated after thermal treatment (baking/roasting). Significant differences in the water content between the samples of the outer toroid and the inner cylinder lasted until the end of the drying process, regardless of the RH of the air. During 96 and 120 h of drying with air at RH60% and RH 80%, it was found that the water content in the samples of the middle and outer toroid was significantly different (*p* ≤ 0.05; Table 3). This proves that water loss from the deeper layers of the sausage bars was slower than from the outer layers [30]. According to Gou et al. [33], one of the main problems when drying meat products is surface hardening or crusting. The crusted surface layer is harder and less permeable than the inner part of the product.

When comparing the water content in the samples of the same bar location of the sausages dried at different air RH, a higher water content was found when air was used at RH 80%, regardless of the drying time considered. However, these differences were not significant (*p* > 0.05, Table 4).

### 3.4. The Content of Basic Chemical Components in KSS Sausages

The average content of basic chemical components (water, protein, fat and salt) in KSS sausages before the drying process was 65.5, 20.0, 11.3 and 1.7%, respectively (Table 4).

As hypothesised, the water content in KSS sausages significantly decreased after the drying process (*p* ≤ 0.05), while the other ingredients significantly increased. According to Olivares et al. [34], the content of protein and fat in meat products during the drying process is concentrated due to water loss. After 96 h of drying with air RH60%, i.e., after achieving the final yield of <70% required by the TSG specification the contents of water, protein, fat, and salt in KSS sausages were 45.6, 31.0, 18.4 and 2.4%, respectively. Sausages dried with air with RH80% after reaching the required yield (i.e., <70% after 120 h of drying) had slightly higher water content and lower protein and fat content that those dried with air at RH60%. However, these differences were not significant (*p* > 0.05). Similar relationships were observed when pork Kabanosy sausages were dried according to the TSG specification [28]. The water content in KSS sausages dried with air at a relative humidity of 80% for 96 h was higher than the water content in the other sausages studied. The higher water content in the above sausages was related to the fact that after 96 h of drying with air at RH80%, they had not yet reached the yield required by the TSG specification (Table 5).

### 3.5. L*, a*, b* Colour Components and TBARS Index of KSS Sausages

The colour components of the products were measured before (0 h) and after 96 and 120 h of the drying process (Table 6). It was found that both the relative air humidity and the drying time had no significant (*p* > 0.05) effect on the values of colour components L*, a*, b* of the cross-sectional area of the KSS sausages. This indicates that the typical colour of KSS sausages was formed after the smoking and baking/roasting process and was not determined by the drying process. The obtained results also show that it is possible to control the relative air humidity (in the range of 60–80%) and the drying time without affecting the colour of the products.

The differentiation in RH of air and drying time had no significant effect (*p* > 0.05) on the values of the TBARS index of the KSS sausages (Table 6). This indicates that it is possible to reduce the relative humidity from 80 to 60% during the drying process without affecting the oxidative changes in the product. Similarly, no significant effect of air relative humidity on the values of TBARS index of dried sausages (kabanosy) was indicated by Chmiel et al. [28].

### 3.6. Sensory Quality of KSS Sausages

There was no significant (*p* > 0.05) influence of RH drying air and drying time on sensory rated desirability of cross-sectional area colour and odour desirability of sausages (Table 7).

However, when evaluating the taste of KSS sausages, significantly lower (*p* ≤ 0.05) scores were found for the KSS sausages air-dried for 96 h with 80% RH compared to other sausages. It should be noted, however, that at this stage of processing, i.e., after 96 h of air drying at 80% RH, the sausages had not yet reached the final yield required by the TSG specification [9]. For the other sausages, the scores in sensory evaluated taste were at a similar level. When analysing the influence of relative air humidity on the evaluated sensory hardness of the sausages, it was found that the product dried with air with higher humidity, i.e., 80% for 120 h, obtained significantly higher (*p* ≤ 0.05) scores in the evaluation of this descriptor. The product dried with air at lower humidity (i.e., 60%) had significantly lower (*p* ≤ 0.05) values for hardness in both sensory evaluation after 96 and 120 h of drying. It also influenced the overall acceptability of the product (Table 7). It was found that KSS sausages dried for 96 or 120 h in air with a relative humidity of 60%, or shorter with air with a relative humidity of 80% performed worse in the overall evaluation than sausages dried with air with a higher humidity (80%) for the time required to reach the yield indicated in the TSG specification, i.e., after 120 h. The obtained results show that a drying process that is too fast, as occurs when using air with lower relative humidity, leads to the formation of a highly dried outer layer of the product with a less dried interior, resulting in the lower overall quality of the product. At the same time, the overall desirability of the product is adversely affected by too short a drying process of the sausages, i.e., 96 h with 80% air relative humidity.

## 4. Conclusions

The use of lower relative air humidity in the process of drying “krakowska sucha staropolska” sausages allows a shorter time scale to be used, for example, 24 h, i.e., by 20%. However, acceleration of the drying process of sausages leads to obtaining a product with very dry outer layers and a less dry interior. Such diversity in the moisture content of the product on its cross-sectional area decreases its overall desirability. Therefore, given the need to produce high-quality products according to the TSG specification, shortening the drying process of sausages by using air with lower relative humidity does not seem desirable, despite the undoubtedly positive economic effect resulting from the shorter drying time.

## Figures and Tables

**Table 1:** Composition of KSS sausages.

Meat and Fat Raw Materials	Share [%]
pork meat class I (ham meat), fat content up to 15%	70
pork meat class IIA (pork shoulder meat), fat content up to 20%	10
pork meat class III (knuckle meat), fat content up to 25%	10
pork backfat	10
Sum	100
Water, additives and spices ^#^	
Water	5
Curing mixture (99.4% NaCl and 0.6% NaNO_2_)	1.5
Black pepper	0.10
White pepper	0.20
Nutmeg	0.10
Fresh garlic	0.40
Sugar	0.20

^#^ For the weight of meat and fat.

**Table 2:** Yield of KSS sausages (mean ± standard deviation).

Feature		Drying Time [h]					
		0 (*n* = 45)	24 (*n* = 21)	48 (*n* = 18)	72 (*n* = 15)	96 (*n* = 12)	120 (*n* = 6)
		Yield [%]					
Relative air humidity during drying	60%	98.8 ^f^ ± 0.3	87.6 ^e,A^ ± 0.1	79.9 ^d,A^ ± 0.7	74.0 ^c,A^ ± 0.4	67.7 ^b,A^ ± 0.4	66.1 ^a,A^ ± 0.6
	80%	98.8 ^f^ ± 0.3	91.6 ^e,B^ ± 2.3	84.4 ^d,B^ ± 1.7	78.0 ^c,B^ ± 1.7	73.0 ^b,B^ ± 1.0	68.0 ^a,B^ ± 0.3

^a, b, c, d, e, f^—the means in the row marked with different letters differ significantly at *p* ≤ 0.05. ^A, B^—the means in the column marked with different letters differ significantly at *p* ≤ 0.05.

**Table 3:** Changes in the water activity of KSS sausages during the drying process (mean ± standard deviation).

Feature		Place of Cutting Out the Sample	Drying Time [h]					
			0 (*n* = 3)	24 (*n* = 3)	48 (*n* = 3)	72 (*n* = 3)	96 (*n* = 3)	120 (*n* = 3)
Relative air humidity during drying	60%	Inner cylinder	0.976 ^b^ ± 0.009	0.964 ^a,b^ ± 0.016	0.963 ^a,b^ ± 0.009	0.960 ^a,b^ ± 0.009	0.952 ^a,b^ ± 0.005	0.950 ^a,B^ ±0.006
		Middle toroid	0.973 ^b^ ± 0.008	0.969 ^a,b^ ± 0.006	0.957 ^a,b^ ± 0.014	0.958 ^a,b^ ± 0.005	0.955 ^a,b^ ± 0.007	0.945 ^a,B^ ± 0.008
		Outer toroid	0.970 ^b^ ± 0.011	0.959 ^a,b^ ± 0.012	0.951 ^a,b^ ± 0.013	0.952 ^a,b^ ± 0.002	0.936 ^a^ ± 0.014	0.932 ^a,A^ ± 0.012
	80%	Inner cylinder	0.976 ^b^ ± 0.009	0.969 ^b^ ± 0.004	0.964 ^b^ ± 0.008	0.962 ^b^ ± 0.012	0.959 ^a,b^ ± 0.010	0.953 ^a,B^ ± 0.006
		Middle toroid	0.973 ^b^ ± 0.008	0.970 ^b^ ± 0.007	0.966 ^b^ ± 0.006	0.963 ^b^ ± 0.010	0.959 ^a,b^ ± 0.007	0.953 ^a,B^ ± 0.002
		Outer toroid	0.970 ^c^ ± 0.011	0.967 ^c^ ± 0.004	0.963 ^b,c^ ± 0.009	0.958 ^a,b,c^ ± 0.005	0.942 ^a,b^ ± 0.017	0.936 ^a,A^ ± 0.005

^a, b, c^—the means in the row marked with different letters differ significantly at *p* ≤ 0.05. ^A, B^—the means in the column for a certain relative air humidity during drying, marked with different letters differ significantly at *p* ≤ 0.05.

**Table 4:** Changes in water content in KSS sausages during the drying process (mean ± standard deviation).

Feature		Place of Cutting Out the Sample	Drying Time [h]					
			0 (*n* = 3)	24 (*n* = 3)	48 (*n* = 3)	72 (*n* = 3)	96 (*n* = 3)	120 (*n* = 3)
Relative air humidity during drying	60%	Inner cylinder	67.2 ^c,B^ ± 1.7	62.9 ^b,c,B^ ± 0.8	59.0 ^b,B^ ± 1.4	55.4 ^a,b,B^ ± 1.4	53.0 ^a,B^ ± 1.9	52.3 ^a,B^ ± 1.4
		Middle toroid	66.3 ^c,A,B^ ± 1.0	60.8 ^b,c,A,B^ ± 1.9	54.6 ^b,A,B^ ± 1.6	50.5 ^a,b,A,B^ ± 1.1	48.1 ^a,B^ ± 1.4	46.6 ^a,B^ ± 1.6
		Outer toroid	64.0 ^c,A^ ± 1.4	56.1 ^b,A^ ± 1.7	50.2 ^b,A^ ± 1.7	46.8 ^a,b,A^ ± 1.2	44.2 ^a,A^ ± 1.7	42.0 ^a,A^ ± 1.8
	80%	Inner cylinder	67.2 ^c,B^ ± 1.7	63.1 ^b,c,B^ ± 0.7	60.7 ^b,B^ ± 0.9	56.9 ^a,b,B^ ± 0.9	55.14 ^a,B^ ± 1.8	53.5 ^a,B^ ± 1.6
		Middle toroid	66.3 ^c,A,B^ ± 1.0	62.5 ^b,c,A,B^ ± 1.5	58.8 ^b,A,B^ ± 1.3	54.6 ^a,b,A,B^ ± 1.2	52.9 ^a,A,B^ ± 1.9	50.6 ^a,B^ ± 2.0
		Outer toroid	64.0 ^c,A^ ± 1.4	59.8 ^b,c,A^ ± 1.1	54.5 ^b,A^ ± 1.2	50.3 ^a,b,A^ ± 1.6	48.0 ^a,A^ ± 2.0	45.6 ^a,A^ ± 1.5

^a, b, c^—the means in the row marked with different letters differ significantly at *p* ≤ 0.05. ^A, B^—the means in the column for a certain relative air humidity during drying, marked with different letters differ significantly at *p* ≤ 0.05.

**Table 5:** Chemical composition of KSS sausages (mean ± standard deviation).

Feature			Content [%]			
			Water	Protein	Fat	Salt
Before drying		0 h (*n* = 3)	65.5 ^C^ ± 1.4	20.0 ^A^ ± 0.7	11.3 ^A^ ± 2.2	1.7 ^A^ ± 0.1
Time and relative air humidity during drying	60%	96 h (*n* = 3)	45.6 ^A^ ± 2.5	31.0 ^B^ ± 2.4	18.4 ^B^ ± 0.6	2.4 ^B^ ± 0.1
		120 h (*n* = 3)	44.4 ^A^ ± 0.5	32.2 ^B^ ± 1.7	18.7 ^B^ ± 0.6	2.5 ^B^ ± 0.1
	80%	96 h (*n* = 3)	50.7 ^B^ ± 1.0	28.5 ^B^ ± 0.7	16.5 ^B^± 0.5	2.4 ^B^ ± 0.1
		120 h (*n* = 3)	47.3 ^AB^ ± 0.9	30.9 ^B^ ± 2.1	17.5 ^B^ ± 0.9	2.4 ^B^ ± 0.1

^A, B, C^—the means in the column marked with different letters differ significantly at *p* ≤ 0.05.

**Table 6:** Colour components L*, a*, b* and TBARS index of KSS sausages (mean ± standard deviation).

Feature			Colour Component			TBARS [mg MAD/kg]
			L*	a*	b*	
Before drying		0 h (*n* = 3)	61.3 ± 4.6	14.1 ± 2.2	4.1 ± 0.5	0.3 ± 0.1
Time and relative air humidity during drying	60%	96 h (*n* = 3)	61.6 ± 2.7	14.6 ± 0.8	3.4 ± 1.0	0.4 ± 0.1
		120 h (*n* = 3)	59.3 ± 1.2	13.4 ± 1.1	3.0 ± 0.4	0.4 ± 0.1
	80%	96 h (*n* = 3)	61.4 ± 2.0	12.8 ± 0.6	3.4 ± 1.4	0.4 ± 0.1
		120 h (*n* = 3)	60.1 ± 1.8	12.4 ± 1.1	2.9 ± 1.1	0.4 ± 0.1

**Table 7:** Sensory quality of KSS sausages (mean ± standard deviation).

Feature			Colour of the Cross-Sectional Area [Point]	Odour [Point]	Taste [Point]	Hardness [Point]	Overall Desirability [Point]
Time and relative air humidity during drying	60%	96 h (*n* = 3)	7.1 ^A^ ± 0.4	7.2 ^A^ ± 0.3	7.4 ^B^ ± 0.2	5.9 ^A^ ± 0.3	6.3 ^A^ ± 0.4
		120 h (*n* = 3)	7.1 ^A^ ± 0.9	7.7 ^A^ ± 0.2	7.1 ^B^ ± 0.4	5.8 ^A^ ± 0.6	6.5 ^A^ ± 0.4
	80%	96 h (*n* = 3)	7.8 ^A^ ± 0.8	7.4 ^A^ ± 0.4	6.2 ^A^ ± 0.2	5.4 ^A^ ± 0.7	6.7 ^A^ ± 0.3
		120 h (*n* = 3)	8.0 ^A^ ± 0.5	7.7 ^A^ ± 0.3	7.7 ^B^ ± 0.5	7.1 ^B^ ± 0.1	7.8 ^B^ ± 0.2

^A, B^—the means in the column marked with different letters differ significantly at *p* ≤ 0.05.

## Data Availability

Data is contained within the article.
